# Islet1 is a direct transcriptional target of the homeodomain transcription factor Shox2 and rescues the Shox2-mediated bradycardia

**DOI:** 10.1007/s00395-013-0339-z

**Published:** 2013-03-01

**Authors:** Sandra Hoffmann, Ina M. Berger, Anne Glaser, Claire Bacon, Li Li, Norbert Gretz, Herbert Steinbeisser, Wolfgang Rottbauer, Steffen Just, Gudrun Rappold

**Affiliations:** 1 Department of Human Molecular Genetics, Institute of Human Genetics, University Heidelberg, INF 366, 69120 Heidelberg, Germany; 2 Department of Internal Medicine II, University of Ulm, Albert-Einstein-Allee 23, 89081 Ulm, Germany; 3 Medical Research Center, University Heidelberg, Theodor-Kutzer-Ufer 1-3, 68167 Mannheim, Germany; 4 Division of Developmental Genetics, Institute of Human Genetics, University Heidelberg, INF 366, 69120 Heidelberg, Germany

**Keywords:** Arrhythmia, Gene regulation, Islet1, Shox2, Sinoatrial node, Transcription factors

## Abstract

The heart’s rhythm is initiated and regulated by a group of specialized cells in the sinoatrial node (SAN), the primary pacemaker of the heart. Abnormalities in the development of the SAN can result in irregular heart rates (arrhythmias). Although several of the critical genes important for SAN formation have been identified, our understanding of the transcriptional network controlling SAN development remains at a relatively early stage. The homeodomain transcription factor Shox2 is involved in the specification and patterning of the SAN. While the *Shox2* knockout in mice results in embryonic lethality due to severe cardiac defects including improper SAN development, Shox2 knockdown in zebrafish causes a reduced heart rate (bradycardia). In order to gain deeper insight into molecular pathways involving *Shox2*, we compared gene expression levels in right atria of wildtype and *Shox2* ^−*/*−^ hearts using microarray experiments and identified the LIM homeodomain transcription factor Islet1 (Isl1) as one of its putative target genes. The downregulation of *Isl1* expression in *Shox2* ^−*/*−^ hearts was confirmed and the affected region narrowed down to the SAN by whole-mount in situ hybridization. Using luciferase reporter assays and EMSA studies, we identified two specific SHOX2 binding sites within intron 2 of the *ISL1* locus. We also provide functional evidence for *Isl1* as a transcriptional target of Shox2 by rescuing the *Shox2*-mediated bradycardia phenotype with *Isl1* using zebrafish as a model system. Our findings demonstrate a novel epistatic relationship between *Shox2* and *Isl1* in the heart with important developmental consequences for SAN formation and heart beat.

**Electronic supplementary material:**

The online version of this article (doi:10.1007/s00395-013-0339-z) contains supplementary material, which is available to authorized users.

## Introduction

During embryogenesis most cells have to become specialized into one of many various cell types that build a whole organism. These patterning and differentiation processes are ensured by the temporal and spatial activation of different transcription factors [50]. Homeobox genes, for example, regulate numerous processes during embryogenesis including heart development. Complex transcriptional regulation networks are important in many aspects of heart cell lineage development and morphogenesis [46].

The cardiac conduction system consists of electrically coupled cardiomyocytes that are responsible for impulse propagation and essential for the rhythmic and coordinated contraction of the heart. A specialized group of cells in the sinoatrial node (SAN) located at the junction of the superior caval vein and right atrium generates the electric impulse to activate the atrial myocardium (also termed primary pacemaker). Together with specialized cells in the atrioventricular node (termed secondary pacemaker) and conduction fibers in the interventricular septum, they form the cardiac conduction system [7, 19]. Abnormalities in the cardiac conduction pathway are responsible for various forms of arrhythmias [41, 44, 58]. Sinus node dysfunction, for example, is associated with sinus bradycardia, atrial tachyarrhythmias, sinus pause or arrest and sinoatrial exit block [10]. Although the morphology, cellular components and electrophysiological properties of the SAN are rather well described [34, 38], knowledge of pathways and transcriptional regulation of the SAN formation are poorly understood. Unraveling the pathways that control pacemaker development and function is of crucial relevance to our understanding of pathologies associated with cardiac conduction.

So far, several transcription factors of the homeobox- and T-box gene families have been shown to be involved in cardiac conduction system specification, patterning, maturation, and function [17, 37]. Among these genes is the paired-related homeodomain transcription factor Shox2, which is known to play a crucial role in early cardiac formation, particularly in SAN development and specification [2, 11, 12, 30, 42]. *Shox2* expression can first be detected in the posterior region of the primitive heart tube at murine embryonic stage E8.5 and as development progresses, *Shox2* is specifically expressed in the sinus venosus myocardium comprising the SAN and the venous valves [2, 12]. These *Shox2* expressing domains develop from myocardium that is added to the venous pole of the developing heart and contributes to the second heart field lineage [5, 52]. Newly recruited myocardium that is added to the arterial pole is divided into the anterior or secondary heart field, while the myocardium added to the venous pole is referred to as the posterior heart field [15]. Loss of function studies revealed an essential role for *Shox2* in the normal anlage of the posterior heart field myocardium [2, 12]. *Shox2* ^−*/*−^ mice die between E11.5 and E17.5 due to heart failure and vascular defects. In particular, the sinus venosus myocardium including the SAN and venous valves show hypoplasia. Moreover, *Shox2* has a crucial role in pacemaker function indicated by severe bradycardia with intermittent sinus exit block after morpholino-mediated knockdown in zebrafish embryos and markedly reduced heart beat rates of isolated *Shox2* ^−*/*−^ hearts [2, 12]. To investigate the molecular processes underlying *Shox2* function, different marker gene expression analyses have been performed. The aberrant expression of *Cx40*, *Cx43*, *Nppa*, and *Nkx2.5* within the SAN of *Shox2* mutant hearts as well as the decreased expression of the SAN-specific marker genes *Tbx3* and *Hcn4* has indicated an abnormal differentiation of pacemaker cells [2, 12]. It has also been shown that *Shox2* prevents the SAN from atrialization by repressing *Nkx2.5* expression [11, 12]. Recently, a further link between *Tbx5*, *Shox2* and *Bmp4* could be established in the developing heart, demonstrating that *Tbx5* signaling in the cardiac pacemaker region controls the expression of *Shox2*, which in turn regulates *Bmp4* in a direct manner [42]. It has also been reported that *Pitx2c* represents an important susceptibility gene for atrial arrhythmias by suppressing left-sided sinus node formation via direct repression of *Shox2* [25, 53]. Taken together, these findings illustrate the importance of *Shox2* function in SAN development and highlight the need for further elucidation of the molecular networks involved.

The aim of our current study was to identify Shox2 target genes during heart development. We used expression analysis to compare the transcriptomes in right atria of wildtype and *Shox2* ^−*/*−^ mouse hearts, and *Islet1* (*Isl1*) was identified as a direct transcriptional target. We have examined putative regulatory elements in the human *ISL1* gene and show a pivotal role for sequences within intron 2 in activating transcription. Furthermore, a functional link between *Shox2* and *Isl1* in vivo was demonstrated using zebrafish as a model by rescuing the *Shox2*-mediated bradycardia via ectopic expression of *Isl1.*

## Methods

### Animals and tissue samples

*Shox2* ^−*/*−^ mice were generated and genotyped as previously described [2]. For maximal reproduction, we crossed our mice into the CD-1 outbred strain. To improve specificity of the microarray analysis, we dissected the right atria of E11.5 hearts that comprise the *Shox2* expressing sinus venosus myocardium and venous valves. For total RNA isolation by TRIzol^®^ (Invitrogen), samples from embryonic right atria (E11.5) of the same genotype (wildtype and *Shox2* ^−*/*−^) were pooled. For whole-mount in situ hybridization, complete hearts were dissected from E11.5 and E12.5 embryos and fixed overnight in 4 % paraformaldehyde at 4 °C.

### Microarray analysis

Gene expression profiling was performed using the GeneChip Mouse Gene 1.0 ST array from Affymetrix (Santa Clara, CA) according to the manufacturer’s protocol. For each genotype (wildtype and *Shox2* ^−*/*−^), RNA from 6 right atria from embryos of 2 independent pregnancies was pooled. Purity and quality of isolated RNA were assessed by the Agilent 2100 Bioanalyzer (Agilent Technologies, Santa Clara, CA) and showed a RIN >9. 200 ng RNA were used for production of end-labeled biotinylated ssDNA and subsequently hybridized to the arrays. Statistical comparisons of chip data based on ANOVA were performed using the software package JMP Genomics, version 4.0 from SAS (SAS Institute, Cary, NC, USA). Briefly, values of perfect-matches were log transformed, quantile normalized and fitted with log-linear models, with probe ID and genotype considered to be constant. Custom CDF version 14 with Entrenz gene-based gene definitions (http://brainarray.mbni.med.umich.edu/Brainarray/Database/CustomCDF/genomic_curated_CDF.asp) different from the original Affymetrix probe set definitions was used to annotate the arrays. The microarray data were deposited in the NCBI GEO database with accession number GSE39924.

### Quantitative RT-PCR

cDNA was synthesized using the SuperScript™ First-Strand Synthesis System for RT-PCR (Invitrogen). Quantitative RT-PCR (qRT-PCR) was performed on a 7500 Fast Real-Time PCR System (Applied Biosystems) using SYBR Green ROX dye (Thermo Scientific). Primer sets used are presented in Supplemental Material (Table S1).

### Generation of plasmid constructs

Cloning of the murine *Isl1* in situ probe, the zebrafish *shox2* and *isl1* expression constructs, the human *SHOX2a* expression construct and generation of the *ISL1* luciferase reporter constructs are described online in the Supplemental Material.

### In situ hybridization

*Shox2* ^−*/*−^ mice have been described previously [2]. Whole-mount in situ hybridization on embryonic mouse hearts was performed as reported [16]. Specific digoxygenin- or fluorescein-labeled RNA probes were as follows: *Isl1* (see Supplemental Material) and *Hcn4* (kindly provided by B. Bruneau, Gladstone Institute of Cardiovascular Disease, USA).

### Immunohistochemistry

E11.5 embryos were fixed in 4 % paraformaldehyde at 4 °C overnight, embedded in OCT and transversely cryosectioned (10 μm). Tbx18 and Hcn4 stainings were enhanced using the ABC (Avidin–Biotin-Complex) reagent from Vector Laboratories (VECTASTAIN Elite Peroxidase ABC-Kit) and the TSA (Tyramide Signal Amplification) system (NEL741, Perkin Elmer) according to the manufacturers’ instructions. For double staining (Isl1/Tbx18 or Isl1/Hcn4), the respective fluorescent secondary antibody was added either during the biotinylated antibody incubation or in a second step afterwards. The following primary antibodies were used: mouse monoclonal antibody against Isl1 (1:100; 39.4D5, Developmental Studies Hybridoma Bank), goat polyclonal antibody against Tbx18 (1:250; C-20, Santa Cruz) and rabbit polyclonal antibody against Hcn4 (1:500; APC-052, Alomone). Alexa Fluor 568 rabbit anti-mouse (1:800; A-11061, Invitrogen), biotinylated horse anti-goat (1:500; BA-9500, Vector Laboratories) and biotinylated goat anti-rabbit (1:500; 550338, BD Pharmingen) were used as secondary antibodies. Nuclei were counterstained with Hoechst (Invitrogen). Imaging was carried out on a Nikon 90i upright epi-fluorescence microscope with a Nikon DS-Qi1 mc camera.

### Cell culture, transfection and luciferase assay

HEK 293 cells were cultured at 37 °C in DMEM medium containing high glucose, supplemented with 10 % fetal calf serum and antibiotics. For luciferase assay analysis, the cells were cotransfected in triplicate with different constructs using polyethylenimine (Sigma-Aldrich). 24 h after transfection, luciferase activity was determined and normalized to Renilla luciferase activity with a dual luciferase assay kit (Promega). Experiments were repeated at least three times in triplicate with consistent results and representative data are shown.

### Electrophoretic mobility shift assay

Electrophoretic mobility shift assay (EMSA) was performed using standard protocols. For the binding reaction, ^32^P-labeled, double stranded *ISL1* oligonucleotides (Oligo1 (+2364/+2418), Oligo2 (+2404/+2458), Oligo3 (+2447/+2502), Oligo4 (+2496/2561); Supplemental Material Table S1), were used together with purified, bacterially expressed recombinant GST-SHOX2 protein [3].

### Zebrafish embryos and microinjections

Care and breeding of zebrafish, *Danio rerio*, were as described previously [55]. For all plasmid and morpholino injection procedures, the TE4/6 wildtype strain was used.

Morpholino-modified antisense oligonucleotides (MO; Gene Tools) were directed against the translational start site (*shox2*-MO [5′-ACGCTGTAAGTTCTTCCATCACTGC-3′]) of zebrafish *shox2*, the splice donor site of exon 2 (*isl1*-MO [5′-TTAATCTGCGTTACCTGATGTAGTC-3′]) of zebrafish *isl1* and the translational start site (*isl1*-MO2 [5′-GATCCCCCATGTCTCCCATGTCAAG-3′] of zebrafish *isl1*. A *shox2*-MO directed against the splice donor site of intron 3 causing the identical bradycardia phenotype has been described previously [2]. *shox2* and *isl1* antisense oligonucleotides or a standard control oligonucleotide (MO-control), diluted in 0.2 mol/liter KCl, were microinjected into one-cell-stage zebrafish embryos [20]. For rescue experiments 0.32 ng of *pDestTol2CG2*-*shox2* or *pDestTol2CG2*-*isl1* was microinjected into one-cell-stage embryos directly after injection of 2.8 ng of *shox2*-MO.

For histology, embryos were fixed in 4 % paraformaldehyde and embedded in JB-4 (Polysciences, Inc). Then, 5-μm sections were cut, dried, and stained with hematoxylin and eosin [43].

For immunoblotting, monoclonal mouse IgG1 anti-rat islet1 antibody (Developmental Studies Hybridoma Bank, DSHB, University of Iowa) and polyclonal anti pan-cadherin (Abcam) were used. Proteins were separated by SDS-PAGE and transferred to polyvinylidene fluoride (PVDF) membrane. Blots were probed with the mentioned antibodies, signals were detected by chemiluminescence (monoclonal anti-rabbit-Hrp and anti-mouse-Hrp) and imaging was performed with Image Quant LAS4000mini (GE Healthcare). Quantification of the immunoblot was performed using the Image Quant TL software.

Whole-mount antisense RNA in situ hybridization was carried out as described [21] using a digoxigenin-labeled antisense probe for zebrafish *shox2* and *islet1*. For immunostaining, zebrafish embryos were fixed in Dent’s fixative and a monoclonal anti-acetylated tubulin antibody (Sigma) was used [40].

### Statistical analysis

Data are presented as mean ± SEM from at least three independent experiments. Comparisons between experimental groups were performed using paired *t* test or 1-way ANOVA. Differences were considered significant if they showed a value of *P* < 0.05.

## Results

### *Shox2* has regulatory effects on *Isl1* expression in the developing sinoatrial node

To identify novel transcriptional targets of Shox2, we performed microarray analysis and compared gene expression levels in right atria of wildtype and *Shox2* ^−*/*−^ hearts. Only right atria comprising the *Shox2* expressing sinus venosus myocardium and venous valves were dissected from murine E11.5 hearts to increase the specificity of the array analysis. Genes were considered to be up- or downregulated if they showed log2 ratios of ≥0.35 or ≤−0.4 with a significance of *P* ≤ 0.0001. Using these selection criteria, we identified 321 genes with significantly altered gene expression (80 up- and 241 downregulated genes). As expected, *Shox2* was among the highest negatively regulated genes. Classification of the 321 genes into gene families using the Gene Set Enrichment Analysis (GSEA) revealed that the highest proportion of regulated genes belongs to the group of transcription factors. Among these, the LIM homeodomain transcription factor Isl1 showed a reduced expression in right atria of *Shox2* ^−*/*−^ embryos.

**Fig. 1:**
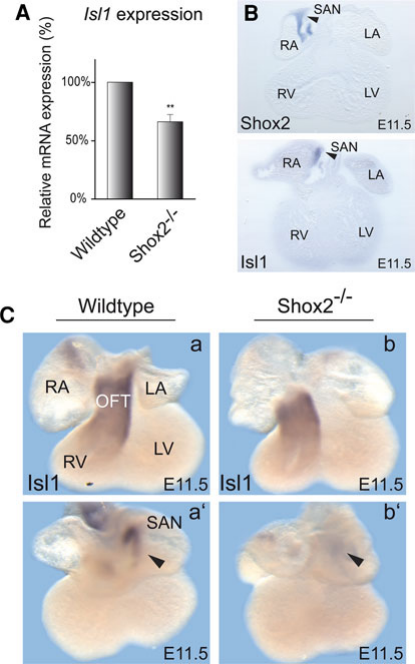
*Shox2* deficiency impairs *Isl1* expression. **A** qRT-PCR analysis using right atrial tissue of E11.5 wildtype and *Shox2* ^−*/*−^ mouse hearts. *Shox2* knockout results in 34 % reduction of *Isl1* mRNA levels in the right atrium of the heart. All results were normalized to *Sdha* (succinate dehydrogenase complex, subunit A) and *Hprt1* (hypoxanthine phosphoribosyltransferase 1) mRNA values. (** *P* = 0.0058; *n* = 21 right atria from 6 different pregnancies and 6 independent experiments). **B** Coexpression of *Shox2* and *Isl1* in the embryonic mouse heart. Sections after whole-mount in situ hybridization on E11.5 wildtype mouse hearts using *Shox2* (*upper section*) and *Isl1* (*lower section*) RNA probes. Murine *Shox2* and *Isl1* are coexpressed specifically in the SAN region of the developing heart (indicated by *black arrowheads*). **C** *Isl1* expression is absent in the SAN of *Shox2* ^−*/*−^ embryos. Whole-mount in situ hybridization on E11.5 wildtype (**a**) and *Shox2* ^−*/*−^ (**b**) mouse hearts using an *Isl1* RNA probe. Both, ventral (**a**, **b**) and dorsal (**a**’, **b**’) views are shown. Murine *Isl1* is expressed in the OFT (**a**) and in the SAN (**a**’) of the developing heart in wildtype embryos. In the *Shox2* ^−*/*−^ mouse heart, *Isl1* expression is still present in the OFT (**b**) but completely absent in the SAN (**b**’), where *Shox2* and *Isl1* expression domains overlap in the wildtype heart. *RA* right atrium, *LA* left atrium, *RV* right ventricle, *LV* left ventricle, *OFT* outflow tract, *SAN* sinoatrial node

To verify *Isl1* as a putative target gene, we carried out qRT-PCR analyses using right atrial tissue samples of wildtype and *Shox2* knockout embryos and confirmed the downregulation of *Isl1* expression in a second independent pool of embryonic heart tissue (Fig. 1A). To further investigate *Isl1* as a putative target gene of Shox2, we compared the spatial expression of both genes and found that *Shox2* and *Isl1* expression overlap in embryonic mouse hearts. *Isl1* expression was detected in the SAN of wildtype hearts, which corresponds to the area where *Shox2* normally is expressed (Fig. 1B). Whole-mount in situ hybridization on isolated E11.5 wildtype and knockout hearts demonstrated that *Isl1* transcripts are completely absent in the SAN region of *Shox2* mutants (Fig. 1C b’), while normal expression was observed in the outflow tract (Fig. 1C b). The lack of *Isl1* expression in the SAN, but unaffected *Isl1* expression in the outflow tract of *Shox2*-null mice could be also confirmed at embryonic stage E12.5 (Fig. S1A). In situ analyses revealed a complete loss of *Isl1* expression in the right atrium (SAN) of knockout hearts, suggesting that the residual *Isl1* expression detected in *Shox2* knockout tissue by qRT-PCR and microarray experiments is likely due to some remaining outflow tract tissue following microdissection. Quantitative expression analysis using outflow tract tissue samples showed high and consistent *Isl1* expression levels in both wildtype and *Shox2* knockout embryos (Fig. S1B).

**Fig. 2:**
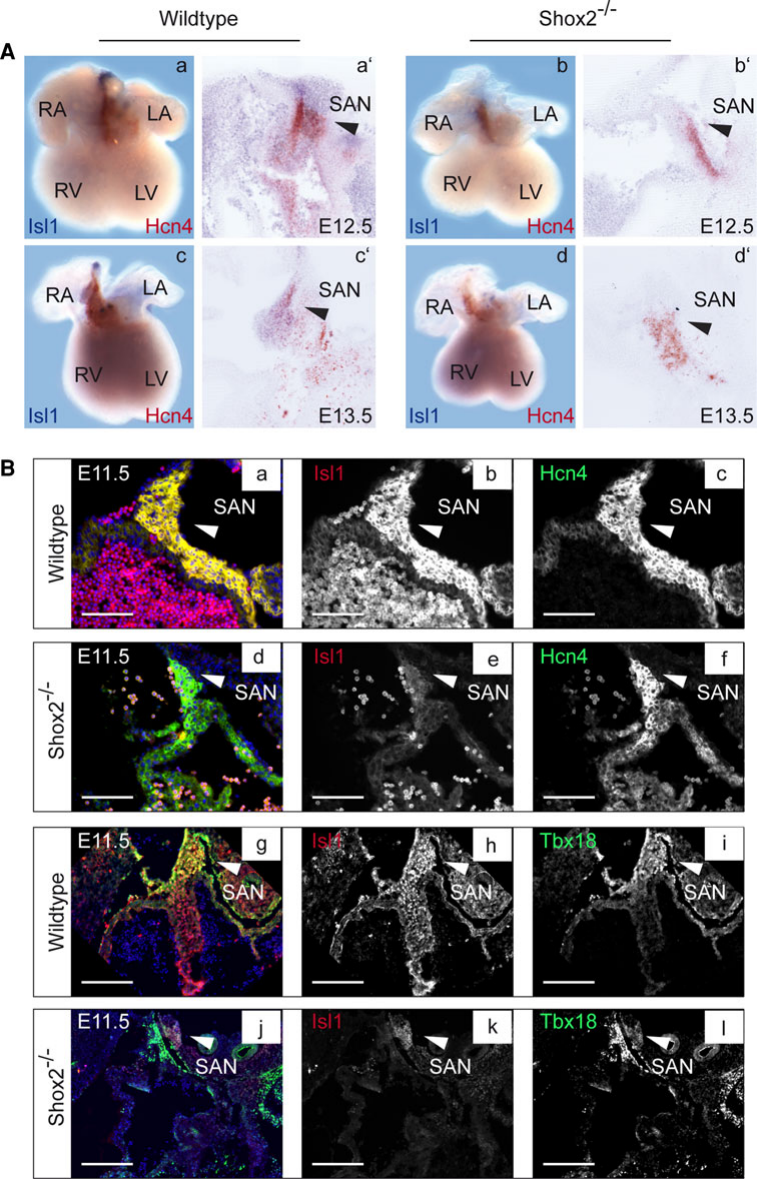
Double staining of wildtype and *Shox2* ^−*/*−^ embryos. **A** Double whole-mount in situ hybridization with corresponding sections (50 μm) on E12.5 (**a**, **b**) and E13.5 (**c**, **d**) wildtype and *Shox2* ^−*/*−^ mouse hearts using *Isl1* and *Hcn4* RNA probes. Murine *Isl1* (*blue* staining) and *Hcn4* (*red* staining) are expressed in the SAN (**a**, **a**’, **c**, **c**’) of the developing heart in wildtype embryos (indicated by *black arrowhead*). In the *Shox2* ^−*/*−^ mouse heart *Isl1* expression is completely absent in the SAN (**b**, **b**’, **d**, **d**’), which represents the overlapping expression domains of *Shox2* and *Isl1* in the wildtype heart. Note that *Hcn4*, another marker of the SAN region, is still expressed in *Shox2* ^−*/*−^ hearts, even if the expression is somewhat reduced (**b**, **b**’, **d**, **d**’). **B** Transverse sections (10 μm) of E11.5 wildtype (**a**–**c**; **g**–**i**) and *Shox2* ^−*/*−^ (**d**–**f**; **j**–**l**) embryos were stained with Isl1 (*red*) and the SAN marker Hcn4 or Tbx18 (*green*). Nuclei are counterstained with Hoechst (*blue*). Isl1 and Hcn4 expression clearly overlaps in the SAN region of wildtype embryos (**a**; *white arrowhead*). Hcn4 is still detectable in the SAN of *Shox2* ^−*/*−^ embryos (**f**), whereas no Isl1 staining is visible in this region (**e**). Isl1 and Tbx18 expression clearly overlaps in the SAN head of wildtype embryos (**g**; *white arrowhead*). Tbx18 is still detectable in the SAN of *Shox2* ^−*/*−^ embryos (**l**), whereas no Isl1 staining is visible in this region (**k**). *RA* right atrium, *LA* left atrium, *RV* right ventricle, *LV* left ventricle, *SAN* sinoatrial node. *Scale bars*: **a**–**f** 1,000 μm; **g**–**l** 500 μm

To validate that the loss of *Isl1* expression in the SAN of *Shox2* knockout hearts is not due to incorrect formation of SAN tissue, we examined the expression of SAN markers in wildtype and knockout hearts using in situ hybridization and immunohistochemistry. While Tbx18 is mainly expressed in the SAN head, Hcn4 is a very prominent marker of the complete developing SAN [45, 56]. Double in situ hybridization experiments using an *Isl1* antisense probe together with *Hcn4* showed coexpression in the SAN of E12.5 and E13.5 wildtype hearts (Fig. 2A a, a’, c, c’). In *Shox2* ^−*/*−^ mouse hearts *Isl1* expression is completely absent in this region, while *Hcn4* transcripts are still detectable (Fig. 2A b, b’, d, d’). Double immunostainings revealed that Hcn4 and Tbx18 colocalize with Isl1 in the SAN of E11.5 wildtype embryos (Fig. 2B a, g). While both markers could be detected in the SAN of wildtype and knockout hearts (Fig. 2B c, f, i, l), Isl1 staining is not detectable in this region in *Shox2* ^−*/*−^ embryos (Fig. 2B e, k). Together, these stainings confirm the loss of Isl1 expression in the SAN of *Shox2* mutants on transcript and protein level at different embryonic stages, while the presence of Hcn4 and Tbx18 confirm that the SAN is formed. The loss of *Isl1* expression is thereby very likely linked to *Shox2* deficiency and does not constitute an indirect effect due to disrupted development of SAN tissue in knockout embryos.

### SHOX2 binds to regulatory elements in an intronic region of the *ISL1* gene

**Fig. 3:**
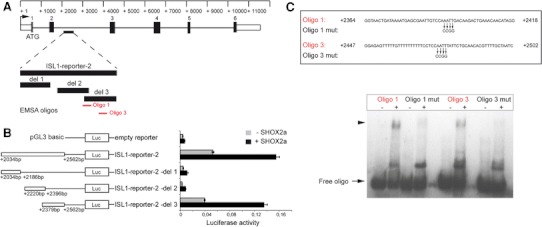
SHOX2 binds to regulatory elements within the *ISL1* gene. **A** Schematic drawing showing the position of the luciferase reporter construct sequences within the genomic region of *ISL1* and the probes generated for EMSA. *Boxes and lines* represent exons and introns, respectively. EMSA probes to which SHOX2 binds are indicated in *red*. **B** SHOX2 increases the transcriptional activity of the *ISL1*-reporter-2, as evaluated by luciferase assay. The responsive reporter element was further narrowed down using deletion (*del*) constructs. HEK 293 cells were transiently cotransfected with the indicated *ISL1* reporter constructs or the empty pGL3 basic vector together with a *SHOX2* expression plasmid or an empty vector (pST-Blue1) and 24 h after transfection, luciferase activity was determined. Experiments were repeated at least three times in triplicate with consistent results and representative data are shown. **C** EMSA revealed specific binding using *Oligo1* and *Oligo3* together with a GST-SHOX2 fusion protein (shifted band is marked by an *arrowhead*). Precise binding sites were identified by the use of mutated probes (*Oligo1* *mut* and *Oligo3* *mut*), with 4 nucleotides exchanged (AATT ↔ CCGG). Free oligo is indicated by an *arrow*. Representative data from three independent experiments with consistent results are shown

To further study the transcriptional control mechanism of *ISL1* by *SHOX2*, we investigated specific binding sites within the *ISL1* locus. As a first step, we performed multispecies conservation analysis for the human *ISL1* locus to identify evolutionary conserved regions. Conservation of DNA sequences provides a route for identifying functional elements in genomes [32]. Sequence comparison of human, frog, chicken, and mouse *Isl1* revealed several highly conserved regions (Fig. S2A). Using luciferase assays, we functionally characterized these highly conserved elements. The reporter constructs used are depicted as black boxes below the conservation plot in Supplemental Material Fig. S2A and are referred to as ISL1-reporter-1 to ISL1-reporter-5. ISL1-reporter-3 and -4 include regulatory elements previously identified downstream of the translational stop codon [22, 23], while ISL1-reporter-1 contains a recently published minimal promoter of the *Isl1* gene [31]. Cotransfections of the different *ISL1* reporter constructs along with a *SHOX2a* expression plasmid revealed a strong increase in luciferase activity only for ISL1-reporter-2, compared to the basal level of the respective reporter alone (Fig. S2B). By transfection of serial gene deletion constructs (genomic locations of the constructs are depicted in Fig. 3A), we confined the *SHOX2* transcriptional regulatory element to a 184-bp region located in the second intron of *ISL1*, corresponding to ISL1-reporter-2del 3 (Fig. 3A, B). To identify the precise SHOX2 binding site, EMSA was performed using four different oligonucleotide probes (Oligo1–Oligo4; Fig. S3A) that cover the respective intronic *ISL1* sequence. We obtained a shift with both Oligo1 (strong binding) and 3 (weaker binding), but not with Oligo2 and 4 (Fig. 3C, Fig. S3B). Competitive EMSAs demonstrated that only high excess (150-fold) of unlabeled Oligo3 (SpC3) competitively affects binding of Oligo1 to SHOX2 (Fig. S4A, left panel), while already a low molar excess (50-fold) of unlabeled Oligo1 (SpC1) competitively inhibits binding of labeled Oligo3 to SHOX2 (Fig. S4A, right panel). Our luciferase assays also show that the sequence encompassing Oligo1 alone is not sufficient to activate *ISL1* by SHOX2 (as this region was also partly included in the ISL1-reporter-2del 2 construct which did not show an increase in luciferase activity upon cotransfection with *SHOX2*) (Fig. 3B), suggesting that both sequences (encompassing Oligo1 and Oligo3) are necessary to activate the *ISL1* gene by SHOX2. Oligo1 and Oligo3 both include AATT-palindromic sequences, which were mutated to CCGG (Oligo1 mut and Oligo3 mut) to confirm sequence specific binding. No shift was obtained using the mutated Oligos, confirming that the AATT motif is essential for SHOX2 binding (Fig. 3C). In addition, competition assays revealed that an excess of unlabeled wildtype Oligos (SpC1 and SpC3) reduce the DNA binding ability of SHOX2, whereas excess of mutant Oligos (MuC1 and MuC3) do not affect the binding (Fig. S4B).

### Bradycardia caused by *shox2* deficiency depends on *isl1* expression

**Fig. 4:**
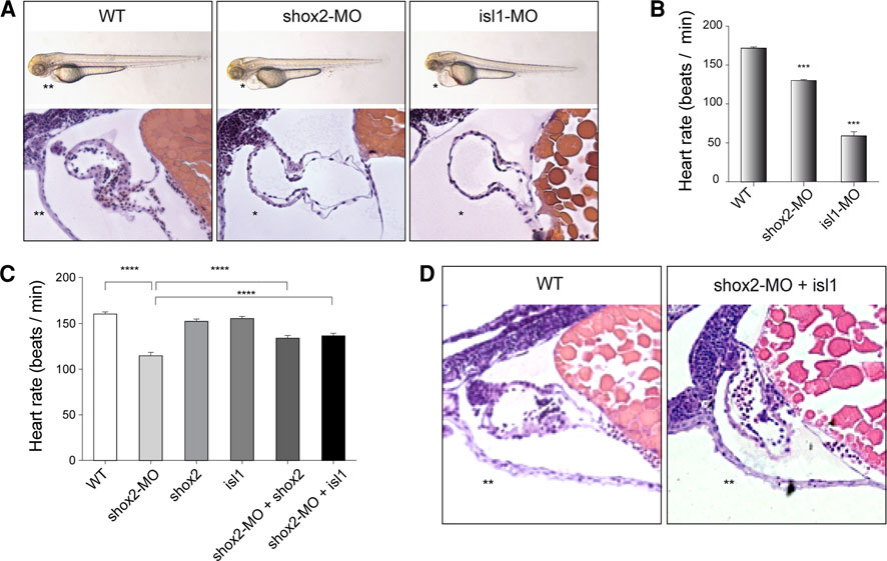
*shox2*-MO-mediated bradycardia can be rescued by *isl1* overexpression. **A** Injection of 2.8 ng *shox2* and *isl1* antisense morpholinos (*shox2*-MO and *isl1*-MO) leads to pericardial edema and pericardial blood congestion due to reduced heart rates in zebrafish embryos. Lateral views of WT, *shox2*-MO and *isl1*-MO injected embryos 72 h post fertilization (hpf). Hematoxylin and eosin staining of sagittal histological sections of WT (*left*), *shox2*-MO (*middle*) and *isl1*-MO (*right*) morphant hearts 72 hpf. Endocardial and myocardial layers of ventricle and atrium are defined, and heart chambers are separated by an atrioventricular ring. **B** Heart rates of *shox2* and *isl1* morphants are decreased by 72 hpf. Injection of *shox2*-MO leads to sinus bradycardia with heart frequencies of 130 beats per min (bpm) compared to WT embryos with 172 bpm. *isl1* deficient embryos exhibit severe sinus bradycardia and frequent pauses in the cardiac contraction with heart frequencies of 59 bmp. (***P < 0.001). **C** *shox2*-MO-mediated bradycardia is partially rescued by cardiomyocyte-specific expression of *isl1*. The mean heart rate of WT embryos 72 hpf amounts to 160 bpm. After *shox2* knockdown (2.8 ng *shox2*-MO) the heart rate is reduced to 114 bpm. Cardiomyocyte-specific overexpression of *shox2* or *isl1* alone (0.32 ng) does not affect the heart beat. Coinjection of 0.32 ng *shox2* or *isl1* plasmid DNA along with 2.8 ng *shox2*-MO results in significantly increased heart frequencies (134 and 137 bmp) compared to *shox2*-MO-injected embryos (114 bpm) at 72 hpf (****P < 0.001; *n* = 32–40 per condition). **D** Hematoxylin and eosin staining of sagittal histological heart sections of WT (*left*) and *shox2*-MO embryos rescued by *isl1* overexpression (*right*) 72 hpf revealed that the rescued hearts, compared to those of *shox2*-MO injected embryos (**A**, *middle picture*), show a phenotype very similar to the WT (*left*). *WT* wildtype, *asterisk* pericardial edema, *double asterisks* no pericardial edema

To functionally assess whether *Shox2* and *Isl1* act in the same genetic pathway, we injected zebrafish embryos with morpholinos (MO). When injecting either 2.8 ng of *shox2*-MO or *isl1*-MO, 82.0 ± 2.6 % (*n* = 175; 2 independent experiments) and 79.1 ± 0.3 % (*n* = 182; 2 independent experiments) of injected embryos, respectively, displayed pericardial edema (Fig. 4A asterisk) and a severely reduced heart rate (Fig. 4B). Embryonic heart morphogenesis proceeds normally in *shox2*-MO and *isl1*-MO injected zebrafish. By 72-h post fertilization (hpf), morphant hearts consist of two heart chambers, atrium and ventricle, with properly developed myocardial and endocardial cell layers. The similarity of phenotypes between *shox2* and *isl1* deficiency in the fish raised the possibility that a close functional interaction between these two genes exists. To address whether the observed cardiac dysfunction caused by *shox2* deficiency could depend on *isl1* function, we examined the ability of *isl1* to compensate the morpholino-mediated loss of shox2 function by transient rescue experiments. DNA constructs expressing *isl1* and *shox2* under the control of the cardiac myosin light chain 2 (*cmlc2*) promoter [18, 33], which drives myocardial-specific expression were used for the rescue experiment. The *cmlc2* promoter also drove expression of GFP, which allowed monitoring of cardiomyocyte-specific expression (Fig. S5A). We coinjected 0.32 ng of the *isl1*-expression construct along with 2.8 ng *shox2*-MO, which was sufficient to induce the bradycardia phenotype and subsequently measured the heart rate of GFP positive embryos after 72 hpf. Myocardial-specific expression of *isl1* could significantly rescue the reduced heart rate in *shox2* morphant embryos. As a control, injection of the *isl1*- or *shox2*-expression construct alone did not cause any changes in embryonic heart rate (Fig. 4C). Histological sections of *shox2*-MO injected hearts rescued by *isl1* overexpression show normal development of the heart without formation of pericardial edema (Fig. 4D), compared to those of *shox2*-MO injected embryos alone (Fig. 4A). In addition, we could show a downregulation of isl1 protein levels in embryos injected with *shox2*-MO (Fig. S5B). Both proteins, shox2 and isl1, are highly expressed during neural development and isl1 plays an important role during differentiation of neurons in the brain. To exclude that shox2 deficiency also affects differentiation of isl1 positive neurons, we analyzed the *isl1* expression after *shox2* knockdown by whole-mount in situ hybridization. *Isl1* expression in the brains of shox2 morphants is indistinguishable from wildtype embryos (Fig. S6A and B). On the other hand, isl1 deficient embryos show a normal expression of *shox2* in the head region (Fig. S6C and D). Furthermore, we performed immunostaining using acetylated Tubulin as neuronal marker (Fig. S6E and F). While the heart rate was reduced after *shox2*-MO injection, no alteration could be detected in neural development. Together, these results indicate an epistatic relationship and a direct functional link between *shox2* and *isl1* in the pacemaking system of zebrafish embryos.

## Discussion

During embryogenesis, the proper development of the cardiac pacemaker is essential for a coordinated heart beat. Defects in the transcriptional network controlling SAN development may cause dysfunction of the primary pacemaker resulting in severe arrhythmias. The elucidation of the molecular mechanisms and key regulators involved in these developmental processes is therefore crucial for our understanding of arrhythmia-related diseases.

The LIM homeodomain transcription factor Isl1 is a prominent marker of second heart field derivatives comprising the right ventricle, outflow tract, inflow tract with the SAN and parts of the atria and plays a crucial role in cardiogenesis [6, 49, 51]. SAN development is initiated in the presence of *Isl1* expressing second heart field-derived cells [35, 48, 51]. Homozygous loss of *Isl1* results in embryonic lethality between E10.5 and E11.0 due to severe cardiac abnormalities. Besides heart looping defects, *Isl1* ^−*/*−^ embryos show hypoplasia or complete absence of the right ventricle, the outflow tract and parts of the atria including the inflow tract [6]. Early in embryogenesis, *Isl1* positive cells, which exhibit multipotent properties [4, 36], contribute to both the arterial (outflow tract) and the venous pole (SAN) of the primary heart tube. Once these progenitor cells differentiate into cardiomyocytes, *Isl1* expression becomes downregulated and almost extinguished from E12.5 to E18.5 [27]. Small numbers of Isl1 positive cells can still be detected in the adult hearts, including the SAN [14, 24, 54].

Although signaling pathways up- and downstream of Isl1 have been investigated in the past [1, 6, 8, 26, 39], little is known about direct regulators controlling the transcriptional activity of this gene in cardiac cells. The only proteins known to regulate *Isl1* expression so far via direct binding are ß-Catenin [28], Forkhead- and Gata4 transcription factors [22, 23] and the POU homeodomain transcription factor Oct1 [31]. While the binding sites for these regulators reside in promoter and enhancer regions up- or downstream of the gene, we could identify a regulatory element within an intronic region of *ISL1*.

**Fig. 5:**
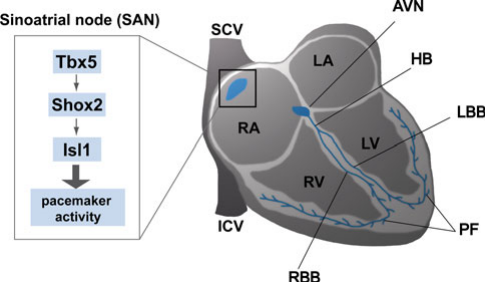
Schematic representation of the epistatic relationship between *Shox2* and *Isl1*. Schematic illustration of the mammalian conduction system highlighting a novel transcriptional regulation in the sinoatrial node. Previously we could show that *Tbx5* regulates *Shox2* in the SAN of the developing heart (12). In the present study, we demonstrate a novel epistatic relationship and a direct functional link between *Shox2* and *Isl1* in this compartment of the heart with important function in the pacemaking system. *AVN* atrioventricular node, *HB* bundle of His, *ICV* inferior caval vein, *LA* left atrium, *LBB* left bundle branch, *LV* left ventricle, *PF* Purkinje fibers, *RA* right atrium, *RBB* right bundle branch, *RV* right ventricle, *SAN* sinoatrial node, *SCV* superior caval vein

In the present study, a connection between the homeodomain transcription factor Shox2 and Isl1 was revealed during cardiac development. We could show that *Shox2* is essential for the expression of *Isl1* in the SAN. Based on luciferase reporter studies and EMSA, our data also demonstrated that SHOX2 directly regulates *ISL1* expression and that this regulation is conducted via the binding to an intronic regulatory element of *ISL1*. To study the transcriptional regulation of *isl1* by *shox2* in vivo, zebrafish was employed as a model. We have shown previously that shox2 loss of function in zebrafish embryos results in severe cardiac dysfunction with sinus bradycardia and intermittent sinus exit block after 72 h of development [2]. Similar data have also been published for *Isl1* [9] and we could confirm these findings experimentally. Furthermore, it has been shown that both genes are expressed at the venous pole of the atrium in zebrafish embryos (48–72 hpf) [2, 57]. In the present study, we demonstrate a functional link between *shox2* and *isl1* in zebrafish embryos by partially rescuing the *shox2*-mediated bradycardia via ectopic expression of *isl1*. Previously, two separate phases of cardiomyocyte differentiation have been shown to exist in zebrafish. The first process, which regulates the differentiation of the venous pole comprising the sinus venosus requires *Isl1* expression, while in a second *Fgf8*-dependent process new cells are added to the arterial pole of the heart [9]. Interestingly, in both *Isl1* mutant fish and *Shox2* knockout mice, *Bmp4* expression is lost in the inflow tract (sinus venosus), but not the outflow tract of the developing heart [9, 42]. In addition, deficiency of both *Isl1* and *Shox2* in fish, as well as *Shox2* deficiency in the mouse results in a bradycardia phenotype [2, 9]. The similarity of the phenotypes observed in *Isl1* and *Shox2* mutants point to a genetic link between these two genes in the development or function of the cardiac pacemaker. Taken together, our findings revealed an interesting interplay between Shox2 and Isl1 in the SAN, which contributes to a novel transcriptional regulation in the cardiac pacemaker system (Fig. 5).

Based on the established functional data, SHOX2 as well as SHOX2 targets represent excellent candidates for human arrhythmias. While genetic variation in *ISL1* is associated with congenital heart disease and cardiomyopathies [13, 47], *SHOX2* has not been linked to any human phenotype yet. In a *SHOX*/*Shox2* replacement mouse model, it was recently shown that a hypomorphic *SHOX* allele is not able to completely rescue the *Shox2* ^−*/*−^ cardiac phenotype. Although the mice survive, they still show arrhythmias, suggesting that *SHOX2* haploinsufficiency may lead to arrhythmia in humans [29]. Thus, it will be interesting to see if both *SHOX2* and *ISL1* are involved in human heart disease, especially arrhythmias.

A possible connection between *Isl1* expressing second heart field-derived cells and the formation of the cardiac conduction system has been previously proposed [35, 48, 54]. As Shox2 directly regulates *Isl1* expression specifically in the embryonic SAN, our data show for the first time a direct functional consequence for *Isl1* in the pacemaking system of the developing heart. Taken together, our data add another piece to the puzzle of regulatory transcriptional networks controlling the development of the pacemaker.

## Electronic supplementary material

Below is the link to the electronic supplementary material.

Supplementary material 1 (DOCX 5717 kb)
