# Supplementary material for: Islet1 is a direct transcriptional target of the homeodomain transcription factor Shox2 and rescues the Shox2-mediated bradycardia
Source: Basic Res Cardiol. 2013 Mar 1;108(2):339. doi: 10.1007/s00395-013-0339-z (PMC3597335; doi:10.1007/s00395-013-0339-z)
Supplement: Supplementary file 1 — Supplementary material 1 (DOCX 5717 kb) [file 395_2013_339_MOESM1_ESM.docx]

Islet1 is a direct transcriptional target of the homeodomain transcription factor Shox2 and rescues the Shox2-mediated bradycardia

Basic Research in Cardiology,

Sandra Hoffmann; Ina M. Berger; Anne Glaser; Claire Bacon; Li Li; Norbert Gretz; Herbert Steinbeisser; Wolfgang Rottbauer; Steffen Just and Gudrun Rappold^*^

^*^Corresponding author

Gudrun Rappold; Institute of Human Genetics; Department of Human Molecular Genetics, INF 366, 69120 Heidelberg; Tel: +49-6221-565059; Fax: +49-6221-565155; E-mail: [Gudrun.Rappold@med.uni-heidelberg.de](mailto:Gudrun_Rappold@med.uni-heidelberg.de)

**SUPPLEMENTAL MATERIAL**

**Detailed Methods**

**Generation of plasmid constructs**

In order to prepare the mRNA probe used for *in situ* hybridization, a specific fragment of *Isl1* DNA sequence (accession number NM_021459) was cloned by PCR using mouse embryonic cDNA E12.5 and the primers Isl1 for/rev (Table S1). The amplified product was then subcloned into the pSTBlue1 vector.

A series of luciferase reporter constructs containing evolutionary conserved regions of the human *ISL1* gene (accession number NM_002202) were amplified from human genomic DNA using different hISL1 Luc primers (Table S1). The amplified PCR products encompassing sequences upstream of the transcriptional start site (+1) and intragenic sequences of *ISL1* were cloned into a *Kpn*I*/Nhe*I digested pGL3 basic vector (Promega): ISL1-reporter-1 (-1784/+472); ISL1-reporter-2 (+2034/+2562); ISL1-reporter-3 (+9913/+14465); ISL1-reporter-2del 1 (+2034/+2186); ISL1-reporter-2del 2 (+2220/+2396); ISL1-reporter-2del 3 (+2379/+2562). The amplified PCR products encompassing downstream sequences of the *ISL1* genomic region were cloned into a *Sac*I/*Nhe*I digested pGL3 promoter vector (Promega): ISL1-reporter-4 (+14160/+17594); ISL1-reporter-5 (+17190/+17790).

To generate the *SHOX2a* expression plasmid, *SHOX2a* cDNA [1] was subcloned into a pT-REx-DEST31 vector (Invitrogen). Zebrafish *shox2* and *isl1* cDNA were subcloned together with a *mlc2*-promotor into the pDestTol2CG2 vector [4] which expresses GFP under the control of the *mlc2*-promotor to generate an expression plasmid for injection in zebrafish embryos.

All constructs were confirmed by sequencing.

**Supplemental Figures and Figure Legends**


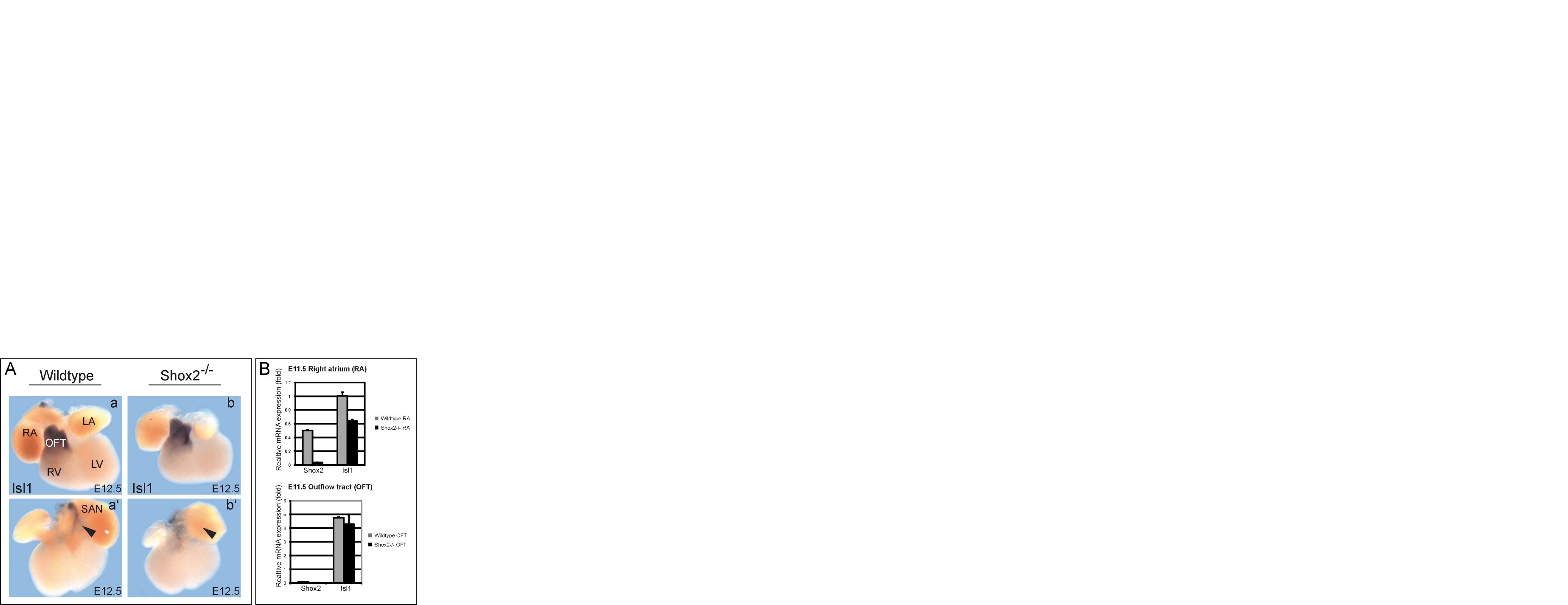


**Figure S1.** ***Isl1* expression is absent in the SAN of *Shox2^-/-^* embryos but unaltered in the outflow tract.** **(A)** Whole mount *in situ* hybridization on E12.5 wildtype (a) and *Shox2^-/-^* (b) mouse hearts using an *Isl1* RNA probe. Both, ventral (a, b) and dorsal (a’, b’) views are shown. Murine *Isl1* is expressed in the OFT (a) and in the SAN (a’) of the developing heart in wildtype embryos. In the *Shox2^-/-^* mouse heart, *Isl1* expression is still present in the OFT (b) but completely absent in the SAN (b’), where *Shox2* and *Isl1* expression domains overlap in the wildtype heart. RA: right atrium, LA: left atrium, RV: right ventricle, LV: left ventricle, OFT: outflow tract, SAN: sinoatrial node. **(B)** *Shox2* and *Isl1* expression in right atria and outflow tract tissue of E11.5 wildtype and *Shox2^-/-^* mouse embryos. *Shox2* is only expressed in the right atrium (RA) of the embryonic heart (upper diagram, left grey bar), whereas *Isl1* expression can be detected in the RA (upper diagram, right grey bar) and even stronger in the outflow tract (OFT; lower diagram, right grey bar). *Shox2* deficiency results in 37% reduction of *Isl1* mRNA levels in the RA (upper diagram, right black bar), whereas the expression of *Isl1* in the OFT (where *Shox2* is not present endogenously) is consistent in wildtype and knockout tissues (lower diagram, right bars). All results were normalized to *Sdha* (*succinate dehydrogenase complex, subunit A*) and *Hprt1* (*hypoxanthine phosphoribosyltransferase 1*) mRNA values.


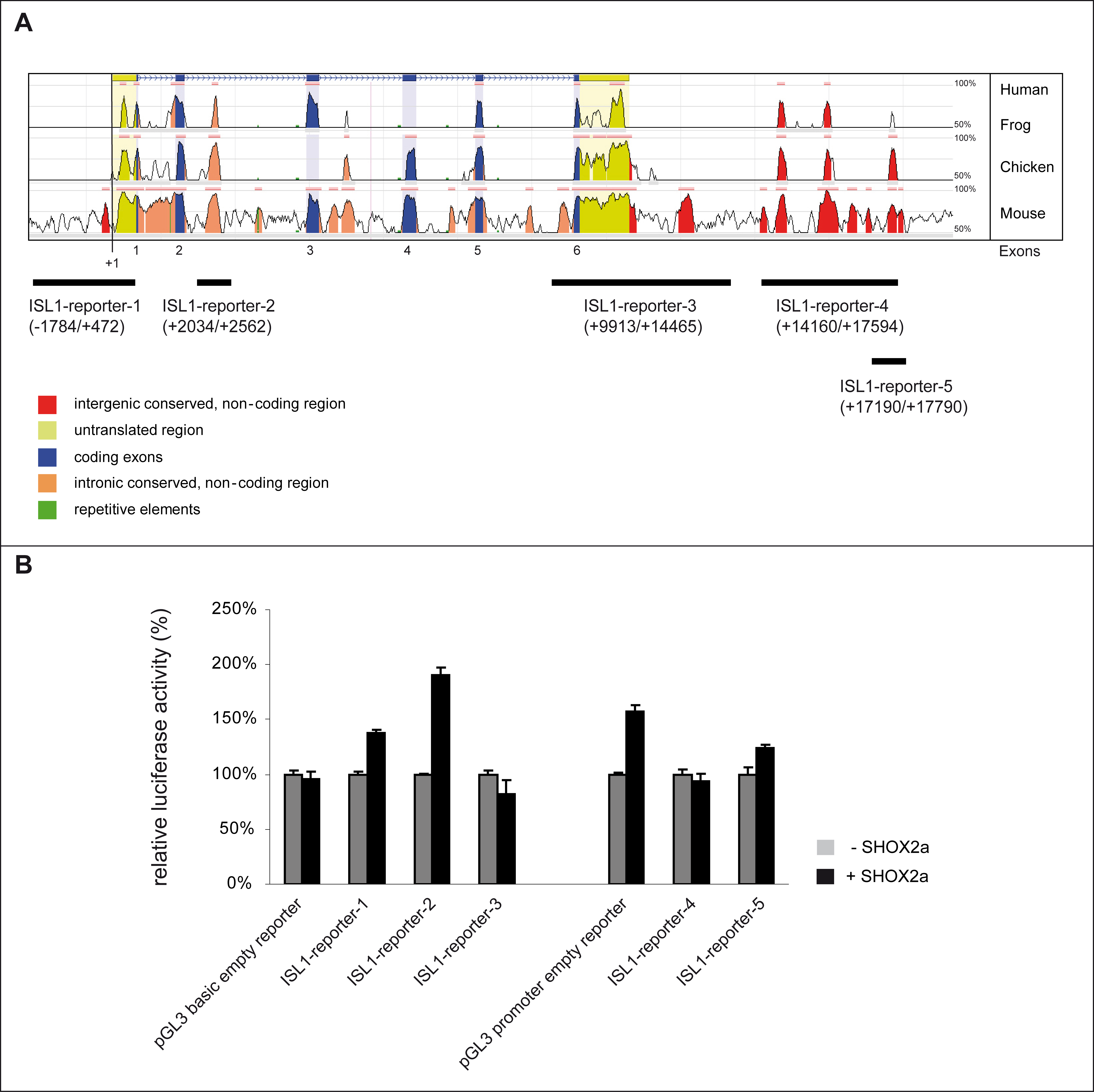


**Figure S2. Evolutionary conserved regions and putative SHOX2 binding sites within the *ISL1* gene (A)** Evolutionary conserved regions of the *ISL1* genomic locus. Multispecies conservation analysis of a 20 kb sequence from the human *ISL1* locus using the ECR browser (conservation throughout human, frog, chicken and mouse of fragments >100bp at 80% identity). Degree of conservation between 50 and 100% is plotted on the X-axis. Generated luciferase constructs according to highly conserved regions are represented in black boxes below the conservation plot (transcriptional start site is indicated by +1). The two non coding elements conserved between human, frog, chicken and mouse contained in ISL1-reporter-3 and ISL1-reporter-4 were previously defined as binding sites for Forkhead transcription factors and Gata4 [2, 3]. **(B)** Luciferase assay using different *ISL1* reporter constructs. HEK 293 cells were transiently cotransfected with a *SHOX2a* expression plasmid (1µg) and different *ISL1* reporter constructs (1µg; genomic locations of the constructs are depicted in A). Highest luciferase activity was measured upon cotransfection of *SHOX2* and *ISL1*-reporter-2.


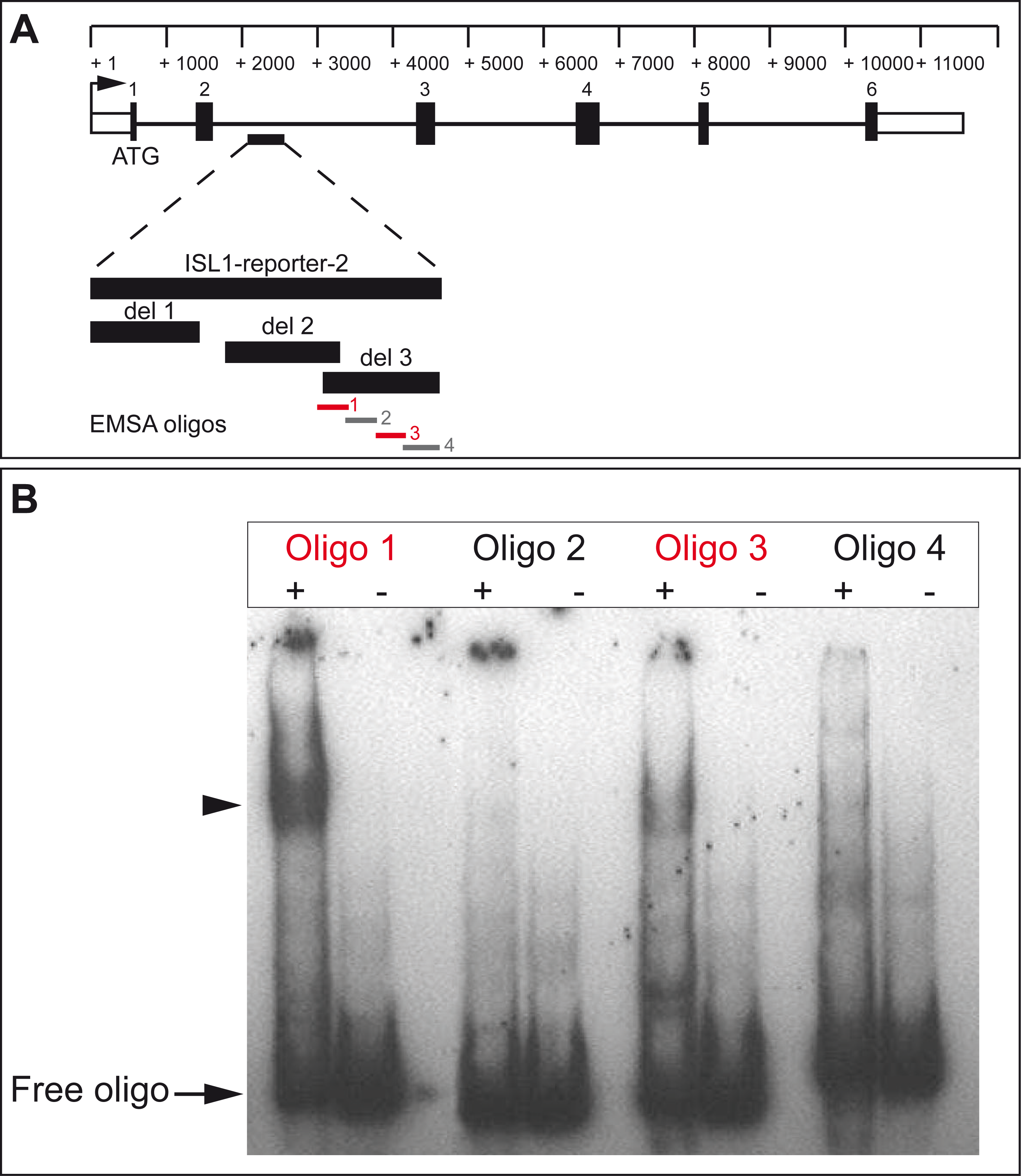


**Figure S3. Identification of SHOX2 binding sites within the *ISL1* gene. (A)** Schematic drawing showing the position of the probes generated for EMSA within the genomic region of *ISL1*. Boxes and lines represent exons and introns, respectively. EMSA probes to which SHOX2 binds are indicated in red. **(B)** EMSA was performed using 4 different radiolabeled double-stranded oligonucleotides to determine the specific binding site of SHOX2 within the *ISL1* gene. The 4 probes (1-4) generated for EMSA cover the sequence of *ISL1*-reporter-2del3 (A), the deletion construct with the highest activation in luciferase assays (Fig. 3B). Shifted bands were observed using Oligo1 (strong) and Oligo3 (weaker) (indicated by arrowhead; see also Fig. 3C).


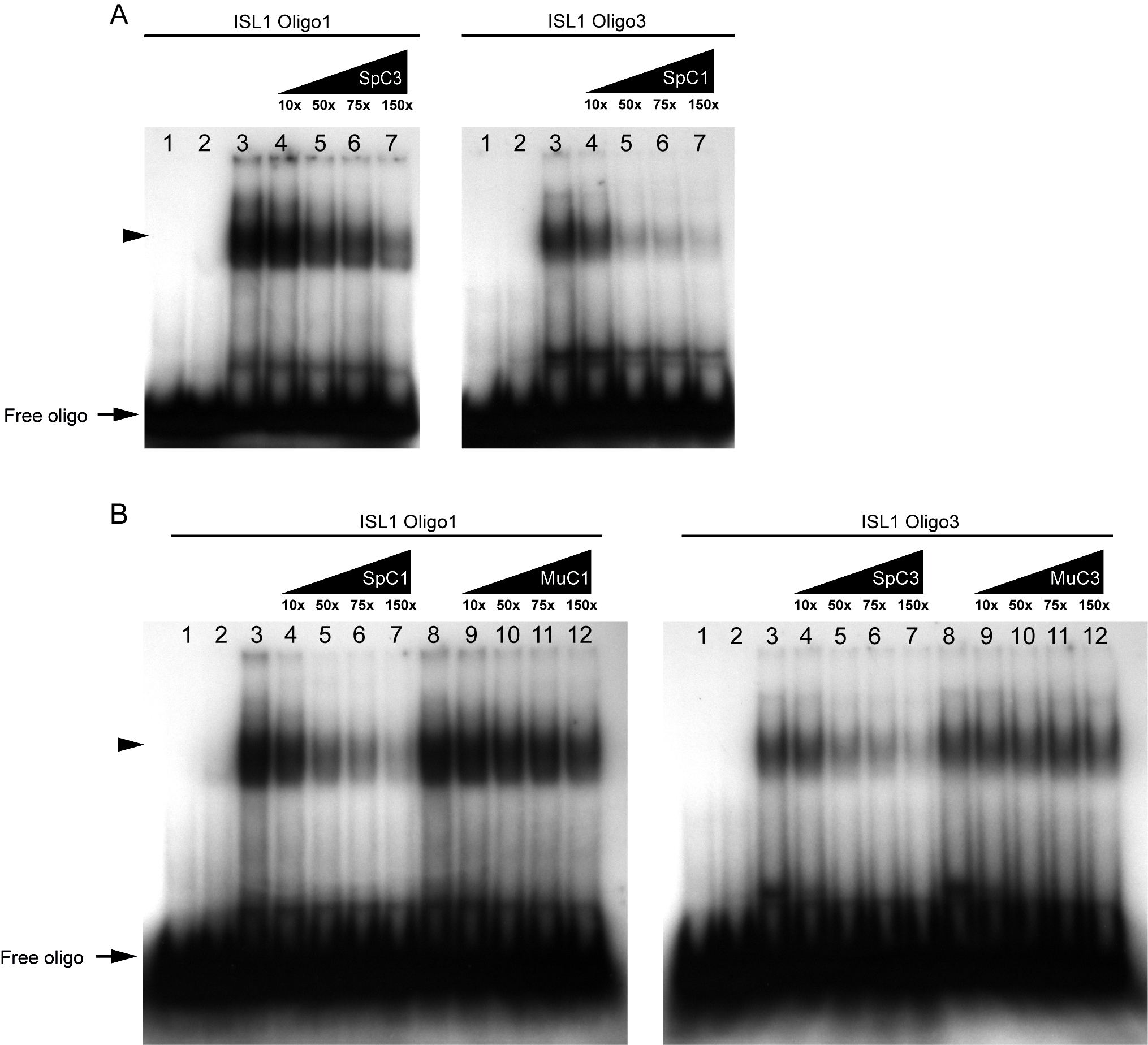


**Figure S4. Competitive Electrophoretic Mobility Shift Assays (EMSAs). (A)** Competition assay using radioactively labeled ISL1 Oligo1 together with unlabeled ISL1 Oligo3 (SpC3) as a specific competitor (left panel) and competition assay using radioactively labeled ISL1 Oligo3 together with unlabeled ISL1 Oligo1 (SpC1) as a specific competitor (right panel). Only high excess (150-fold) of unlabeled Oligo3 competitively inhibits binding of SHOX2 to ISL1 Oligo1 (left panel), whereas already a low molar excess (50-fold) of unlabeled Oligo1 (SpC1) affects the binding of SHOX2 to ISL1 Oligo3 (right panel). 1: free oligonucleotide (radioactively labeled Oligo); 2: GST alone; 3 GST-SHOX2; 4-7: 10-fold, 50-fold, 75-fold and 150-fold molar excess of specific competitor (non-radioactively labeled Oligo). Shifted band is marked by an arrowhead; free oligonucleotide is indicated by an arrow. **(B)** Competition assay using radioactively labeled ISL1 Oligo1 together with unlabeled ISL1 Oligo1 (SpC1) as a specific competitor and unlabeled mutated ISL1 Oligo1 (MuC1) as an unspecific competitor (left panel). Competition assay using radioactively labeled ISL1 Oligo3 together with unlabeled ISL1 Oligo3 (SpC3) as a specific competitor and unlabeled mutated ISL1 Oligo3 (MuC3) as an unspecific competitor (right panel). In both assays an excess of non-radioactively labeled oligonucleotide (SpC) reduces the DNA binding ability of SHOX2 (shifted band is marked by an arrowhead), whereas excess of a mutated oligonucleotide (MuC) does not affect DNA binding. Free oligonucleotide is indicated by an arrow. 1: free oligonucleotide; 2: GST alone; 3 GST-SHOX2; 4-7: 10-fold, 50-fold, 75-fold and 150-fold molar excess of SpC (non-radioactively labeled ISL1 Oligo); 8: GST-SHOX2; 9-12: 10-fold, 50-fold, 75-fold and 150-fold molar excess of mutated competitor; MuC (non-radioactively labeled mutated ISL1 Oligo).


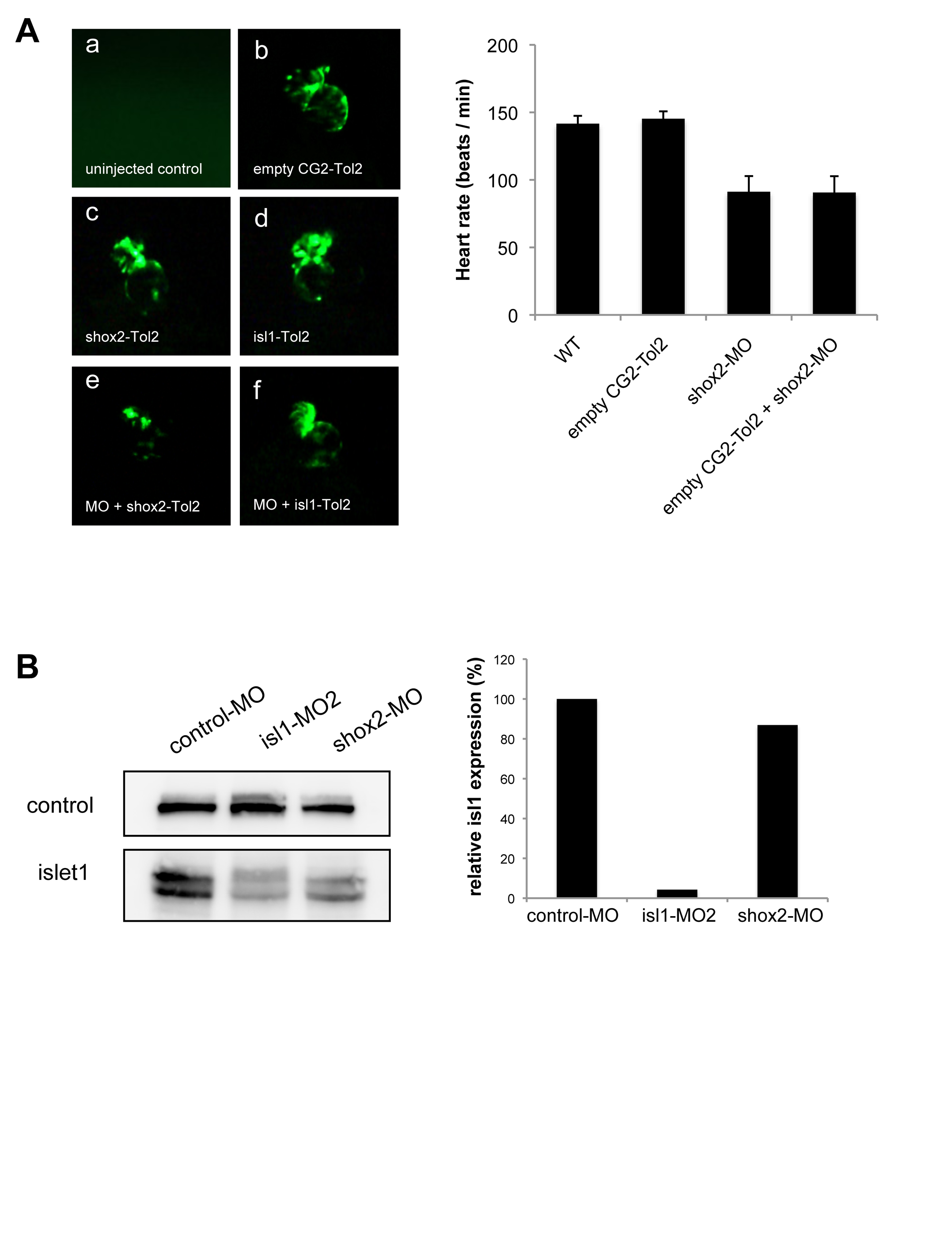


**Figure S5. (A)** Coinjection of *shox2* and *isl1* expression constructs together with *shox2*-MO into zebrafish embryos. Monitoring of cardiomyocyte-specific expression of *shox2* (c, e) and *isl1* (d, f) expression constructs via coexpression of GFP. The empty expression vector does not influence the heart rate of zebrafish embryos 72 hpf (right panel). **(B)** isl1 protein levels after *isl1*-MO2 and *shox2*-MO injection into zebrafish embryos 72 hpf. Western blot (left) using pan-Cadherin as control antibody (upper blot) and isl1 as specific antibody (lower blot) following quantitative analysis (right) indicate decreased levels of isl1 protein in *shox2*-MO injected embryos.


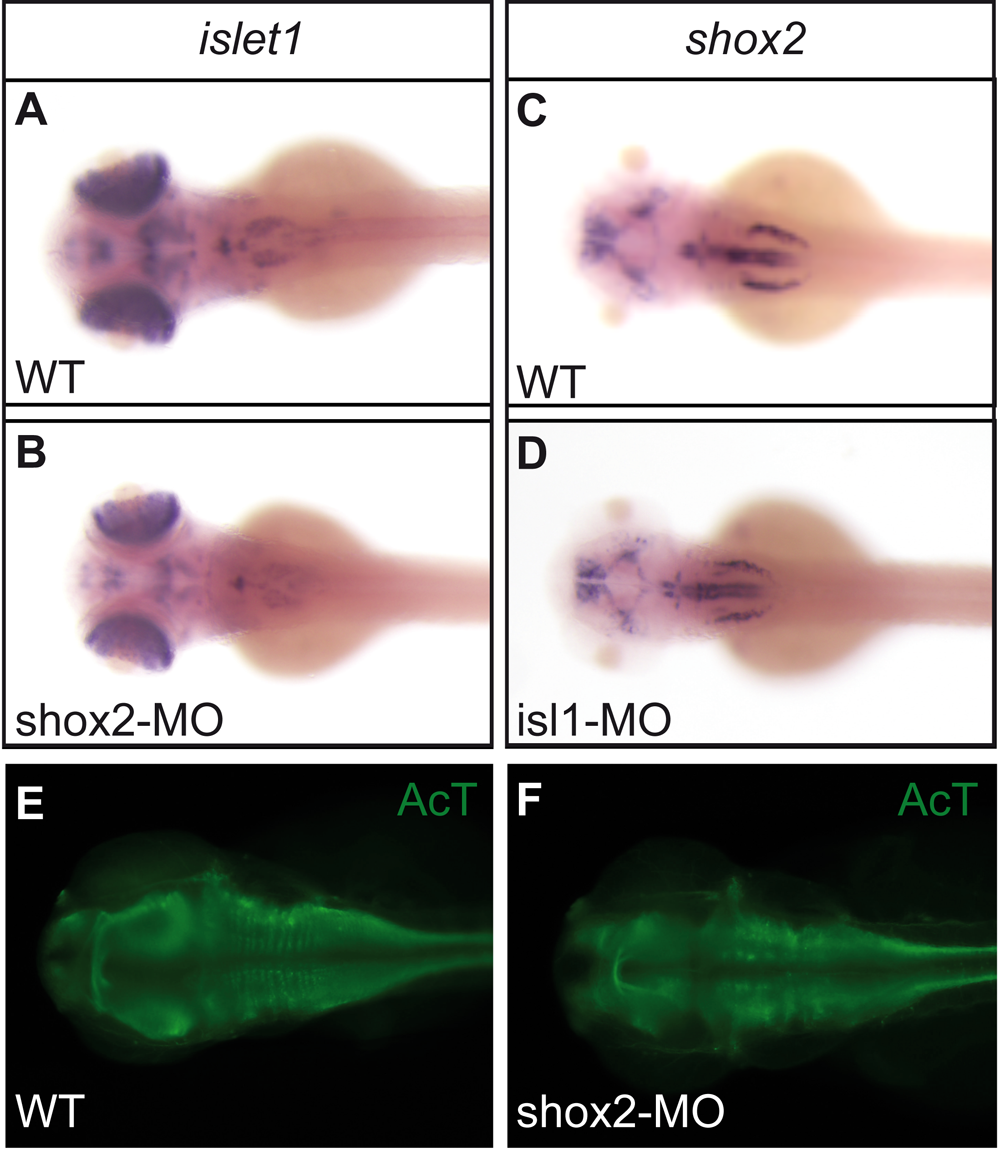


**Figure S6. Visualization of neuronal development in *shox2*-MO and *isl1*-MO injected zebrafish embryos.** Whole mount *in situ* hybridization on *shox2*-MO (B) and *isl1*-MO (D) injected zebrafish embryos 72h hpf using *islet1* (A, B) and *shox2* (C, D) RNA probes. The expression of both genes is the same in wildtype (WT) and morpholino (MO) injected embryos during neural development, indicating that *shox2* deficiency does not affect differentiation of isl1 positive neurons. Immunostaining of *shox2*-MO injected embryos using acetylated Tubulin (AcT) as neuronal marker (E, F) confirm normal neural development.

**Supplemental Tables**

| **Table S1.** Oligonucleotides | | | | | |
| --- | --- | --- | --- | --- | --- |
| **Name** | **Sequence (5' - 3')** | | **T_M_ (°C)** | **Product size (bp)** | **Application** |
| mHprt1 qRT for | TCC TCC TCA GAC CGC TTT T | | 60 | 90 | qRT-PCR |
| mHprt1 qRT rev | CCT GGT TCA TCA TCG CTA ATC | |  |  |  |
| mSdha qRT for | Cat gcc agg gaa gat tac aaa | | 60 | 88 |  |
| mSdha qRT rev | Gtt ccc caa acg gct tct | |  |  |  |
| mShox2 qRT for | ACC AAT TTT ACC CTG GAA CAA C | | 60 | 141 |  |
| mShox2 qRT rev | TCG ATT TTG AAA CCA AAC CTG | |  |  |  |
| mIsl1 qRT for | gca acc caa cga caa aac taa | | 60 | 78 |  |
| mIsl1 qRT rev | cca tca tgt ctc tcc gga ct | |  |  |  |
| mIsl1 for | CAG CAA GAA CGA CTT CGT GA | | 60 | 720 | Cloning |
| mIsl1 rev | GGA CTG GCT ACC ATG CTG TT | |  |  |  |
| hISL1 Luc1 for | ggg ggt acc ctg gtt tga agg agg atg ga | | 60 | 4553 |  |
| hISL1 Luc1 rev | ccc gct agc AGC TGT GGC TAA GTG GGA AA | |  |  |  |
| hISL1 Luc2 for | ggg ggt acc tgg tgg cat caa tgc aat ag | | 60 | 529 |  |
| hISL1 Luc2 rev | ccc gct agc ggc ttt cta agg cag aaa aca a | |  |  |  |
| hISL1 Luc3 for | ggg ggt acc gga gcc tcc tct caa tct cc | | 60 | 4553 |  |
| hISL1 Luc3 rev | ccc gct agc aaa gcc aca tgg gat ttt aca | |  |  |  |
| hISL1 Luc4 for | ggg gag ctc cct ttg aaa caa ggt ttc tgg | | 60 | 3435 |  |
| hISL1 Luc4 rev | ccc gct agc cct tta aaa gtt aat tcc cac ca | |  |  |  |
| hISL1 Luc5 for | ggg ggt acc tgg tta tgt gcc taa cgt ggt | | 60 | 601 |  |
| hISL1 Luc5 rev | ccc gct agc cga gaa tat gag taa tta aaa tca gca | |  |  |  |
| hISL1 Luc2 del1 for | ggg ggt acc tgg tgg cat caa tgc aat ag | | 60 | 153 |  |
| hISL1 Luc2 del1 rev | ccc gct agc tcc aag gaa tat tta ggt tgg ttt | |  |  |  |
| hISL1 Luc2 del2 for | ggg ggt acc aac cct ttg gtg aca tct aac tg | | 60 | 177 |  |
| hISL1 Luc2 del2 rev | ccc gct agc ttt gga caa ttc gct cat tt | |  |  |  |
| hISL1 Luc2 del3 for | ggg ggt acc gag cga att gtc caa att ga | | 60 | 184 |  |
| hISL1 Luc2 del3 rev | ccc gct agc ggc ttt cta agg cag aaa aca a | |  |  |  |
| ISL1 EMSA for1 | Oligo1 | ggg ggt aac tga taa aat gag cga att gtc caa att gac aag act gaa aca aca tag g | | | EMSA |
| ISL1 EMSA rev1 |  | ggg cct atg ttg ttt cag tct tgt caa ttt gga caa ttc gct cat ttt atc agt tac c | | |  |
| ISL1 EMSA for2 | Oligo2 | ggg ctg aaa caa cat agg aac ttt ctg agt ttg gtt ttg ttg ttt tgg aga gtt ttt g | | |  |
| ISL1 EMSA rev2 |  | ggg caa aaa ctc tcc aaa aca aca aaa cca aac tca gaa agt tcc tat gtt gtt tca g | | |  |
| ISL1 EMSA for3 | Oligo3 | ggg gga gag ttt tgt ttt ttt ttt cct cca att tat tct gca aca cgt ttt gct aat c | | |  |
| ISL1 EMSA rev3 |  | ggg gat tag caa aac gtg ttg cag aat aaa ttg gag gaa aaa aaa aac aaa aac tct cc | | |  |
| ISL1 EMSA for4 | Oligo4 | ggg gct aat ctc aag ttt cct ctg act tgt gtg tat gta tca gaa act ttg ttt tct gcc tta gaa agc c | | |  |
| ISL1 EMSA rev4 |  | ggg ggc ttt cta agg cag aaa aca aag ttt ctg ata cat aca cac aag tca gag gaa act tga gat tag c | | |  |
| ISL1 EMSA for1 mut | Oligo1 mut | ggg ggt aac tga taa aat gag cga att gtc cac cgg gac aag act gaa aca aca tag g | | |  |
| ISL1 EMSA rev1 mut |  | ggg cct atg ttg ttt cag tct tgt ccc ggt gga caa ttc gct cat ttt atc agt tac c | | |  |
| ISL1 EMSA for3 mut | Oligo3 mut | ggg gga gag ttt ttg ttt ttt ttt tcc tcc ccg gta ttc tgc aac acg ttt tgc taa tc | | |  |
| ISL1 EMSA rev3 mut |  | ggg gat tag caa aac gtg ttg cag aat acc ggg gag gaa aaa aaa aac aaa aac tct cc | | |  |

**Supplemental References**

1. Blaschke RJ, Monaghan AP, Schiller S, Schechinger B, Rao E, Padilla-Nash H, Ried T, Rappold G (1998) SHOT, a SHOX-related homeobox gene, is implicated in craniofacial, brain, heart, and limb development. Proc Natl Acad Sci USA 95:2406-2411 doi:10.1073/pnas.95.5.2406

2. Kang J, Nathan E, Xu SM, Tzahor E, Black BL (2009) Isl1 is a direct transcriptional target of Forkhead transcription factors in second-heart-field-derived mesoderm. Dev Biol 334:513-522 doi:10.1016/j.ydbio.2009.06.041

3. Kappen C, Salbaum JM (2009) Identification of regulatory elements in the Isl1 gene locus. Int J Dev Biol 53:935-946 doi:10.1387/ijdb.082819ck

4. Kwan KM, Fujimoto E, Grabher C, Mangum BD, Hardy ME, Campbell DS, Parant JM, Yost HJ, Kanki JP, Chien CB (2007) The Tol2kit: a multisite gateway-based construction kit for Tol2 transposon transgenesis constructs. Dev Dyn 236:3088-3099 doi:10.1002/dvdy.21343
